# Patient Expectations of Bariatric Outcomes, Baseline, and Long-term Evaluation

**DOI:** 10.1007/s11695-025-07997-0

**Published:** 2025-07-04

**Authors:** Lindsy van der Laan, Ellen A. M. Kuipers, Josien G. Timmerman, Mirjam A. Kaijser, Marc J. van Det, Marloes Emous

**Affiliations:** 1 Center for Obesity Northern-Netherlands (CON), Department of Metabolic Bariatric Surgery, Medical Center Leeuwarden, Leeuwarden, Netherlands; 2 University of Groningen, University Medical Center Groningen, Groningen, Netherlands; 3 Department of Surgery, Hospital Group Twente Almelo/Hengelo, Almelo, Netherlands; 4 Biomedical Signals and Systems, Faculty of Electrical Engineering, Mathematics and Computer Science, University of Twente, Enschede, Netherlands; 5 ZGT Academy, Hospital Group Twente Almelo/Hengelo, Almelo, Netherlands

**Keywords:** Expectations, Metabolic Batriatric Surgery, Complications, Physical Domain, Phychological Domain, Social Domain, Motivation, Informed Decision-making, Postoperative Satisfaction

## Abstract

**Background:**

This study investigates preoperative patient expectations of metabolic bariatric surgery (MBS), focusing on anticipated challenges and effects on physical, social, and psychological domains.

**Methods:**

A prospective, cross-sectional multicenter survey among adult patients awaiting primary MBS was conducted. The questionnaire gathered data on (1) the top three reasons for seeking surgery, (2) anticipated weight loss, and (3) expected remission of obesity-related diseases. Patients also rated the importance of being informed about potential complications, anxiety over complications, and the extent to which these complications influence their decision to undergo surgery. Additionally, patients reported their expectations of physical, social, and psychological changes.

**Results:**

A total of 242 patients completed the questionnaire. Patients prioritized mobility and health as the main reasons for choosing MBS, with other key factors including pain reduction, remission of obesity-related diseases, and physical fitness. Patients expected a median percentage total weight loss of 32% and a percentage excess weight loss of 81%. Most patients expected total remission of obesity-related diseases. Although patients valued information on potential complications, these risks did not induce anxiety or affect the decision to undergo MBS. The most anticipated physical changes included improved mobility, pain reduction, and better health/fitness. The most expected social and psychological changes were related to self-image and emotional well-being.

**Conclusions:**

Improved mobility and health were the main reasons for undergoing MBS. Although participants had high weight loss expectations, their estimations for obesity-related diseases resolutions were accurate. Despite prioritizing information about potential complications, this did not discourage patients from choosing MBS.

**Supplementary Information:**

The online version contains supplementary material available at 10.1007/s11695-025-07997-0.

## Introduction

Metabolic bariatric surgery (MBS) is an effective and durable treatment option for patients with severe obesity in terms of weight loss and improvement of obesity-related diseases [1]. Traditionally, most studies examining outcomes after MBS have primarily focused on weight loss and the reduction of obesity related diseases as the main outcomes of interest [1–5]. However, a growing body of research is shifting the focus toward quality of life, acknowledging that MBS is an invasive intervention with a broad impact on various aspects of a patient’s life, including mental health, nutritional habits, and overall well-being [2, 6–12]. Keeping that in mind, patients have various reasons for undergoing MBS, beyond just physical health concerns [13–15]. To provide realistic information before MBS, it is important to gain knowledge about the patient’s motivation for having surgery as well as their expectations of MBS.

Previous literature has demonstrated that the primary motivation for patients seeking MBS is related to physical health, such as weight loss and improvement of obesity-related diseases [16–20]. In addition, a variety of other motivations were mentioned, including increasing fertility, pain reduction, and improving physical activity, social life, and emotional well-being [15, 20, 21]. Remarkably, Hult et al. showed that the most common preoperative motivation for undergoing MBS, i.e., weight loss, differed from the most common postoperative source of satisfaction, which was improved self-esteem [14]. Nonetheless, weight loss remained one of the three items that participants were most satisfied with after surgery.

Existing studies do show that patients have unrealistic goals in terms of weight loss after MBS [16, 20–22]. Expected excess weight loss percentages (%EWL) between 71 and 94% have been reported [16, 20–22], while 18 studies of gastric bypass surgery showed a weighted mean %EWL of 57% in the long term (≥ 10 years) [23]. Moreover, given that an EWL of at least 50% is defined as an optimal clinical response after surgery, presurgical weight loss expectations are high [23, 24].

Despite substantial progress in characterizing patients’ preoperative expectations and motivations for MBS, limited attention has been given to their perspectives on potential postoperative challenges and difficulties. Addressing this gap is important, as such concerns may contribute to long-term satisfaction after surgery. Van Rijswijk et al. concluded that patients accept substantial risks of short-term complications (≤ 30 days after surgery), acute internal herniation (≥ 30 days), and mortality (≤ 30 days) to achieve their weight loss objectives [25]. Patients with a higher body mass index (BMI) accepted higher risks of complications and mortality within 30 days, compared to patients with a lower BMI. Notably, the influence of age on the willingness to accept substantial risks remains unexplored [25]. Furthermore, no attention has been given to other potential problems, barriers, or challenges in the longer term after surgery.

Therefore, the aim was to extend earlier work by exploring patients’ main expected concerns, barriers, and challenges in the long term after MBS, as well as their preoperative expectations of physical, social, and psychological aspects. In addition, potential differences in subgroups based on gender, BMI, age, and the presence of obesity-related diseases were assessed. Insight into patients’ preoperative expectations may help in preoperative counseling, managing patients’ expectations, and may have important implications in prioritizing patient-centered outcomes.

## Methods

### Study Population

A cross-sectional multicenter survey in adult patients awaiting primary MBS was conducted. Two non-academic teaching hospitals in the Netherlands participated: Medical Center Leeuwarden (MCL) and Hospital Group Twente Almelo/Hengelo (ZGT). All patients who were approved for primary MBS were asked to participate in this study between April and October 2023. Patients not fluent in Dutch or illiterates were excluded because of the inability to understand and complete the Dutch questionnaire.

The medical ethical committee approved the study (RTPO Leeuwarden, nWMO 2023 0030; ZGT 2023–13 nWMO) and informed consent was obtained from all patients.

### Study Procedure

All patients complied with the implemented International Federation for the Surgery of Obesity and Metabolic Disorders (IFSO) guidelines for undergoing MBS from 2014: BMI ≥ 40 kg/m^2^ or BMI ≥ 35 kg/m^2^ with at least one obesity-related disease [26]. Patients attended an information session on MBS before their preoperative consultation with a surgeon specializing in MBS, as per standard care protocol. The most commonly performed procedures at both hospitals include the one anastomosis gastric bypass, the Roux-en-Y gastric bypass, and, to a lesser extent, the sleeve gastrectomy. During the preoperative consultation, patients were invited to participate in the study by either the MBS nurse or the surgeon. Upon agreement, they were provided with a questionnaire on paper, including further explanation of the study. Returning the completed questionnaire signified informed consent, as stated in the introduction of the questionnaire.

### Questionnaire

A questionnaire in Dutch (the patients’ native language) was developed based on literature and clinical expertise (Appendix 1). The following demographic variables were included: gender, age, body weight, length, highest education level, and obesity-related diseases. The top three reasons for seeking surgery and patients’ expectations of MBS regarding weight loss and changes in physical, social, and psychological domains were assessed using open-ended questions. Answer options regarding the remission of obesity-related diseases were no remission (no changes in treatment or pain complaints), partial remission (less treatment or less pain complaints), total remission (no longer requiring treatment or no pain complaints anymore), and deterioration (more treatment or more pain complaints).

The impact of short-term (≤ 30 days after surgery) and long-term complications (> 2 years after surgery) was assessed in three domains: (1) the importance of being informed about the complication during the preoperative period, (2) the anxiety of developing the complication, and (3) the impact of the complication on the consideration of undergoing MBS. For every complication, patients were asked to rate these three domains on a 5-point Likert scale. Additionally, patients were given the opportunity to express fears about unaddressed issues.

### Statistical Analysis

Baseline characteristics were expressed by descriptive statistics. Parametric data were presented as mean ± standard deviation (SD), nonparametric data as median with interquartile range (IQR), and categorical data as frequencies and percentages. Expected weight loss was expressed as %EWL and percentage total weight loss (%TWL). Subgroup analyses were conducted for expected weight loss, expected remission of obesity-related diseases, and the impact of complications for four groups based on (1) gender (men versus women), (2) BMI (BMI < 50 kg/m^2^ versus BMI ≥ 50 kg/m^2^), (3) age (< 30 years versus > 50 years), and (4) presence of obesity-related diseases (with versus without obesity-related diseases). Nonparametric data were analyzed using the Mann–Whitney *U* test. Categorical data were analyzed with a chi-square test. To account for multiple comparisons in the subgroup analyses related to the impact of complications, *p*-values were adjusted using the Benjamini–Hochberg procedure. A *p*-value of < 0.05 was considered statistically significant. Statistical analysis was performed using SPSS Statistics (version 28, SPSS Inc., Chicago, IL, USA).

Qualitative data from open-ended questions were analyzed inductively, with categories generated by two researchers (L.v.d.L. and E.K.) and discussed with the other authors. The frequency and the percentages of the subsequent categories were reported.

## Results

### Patient Characteristics

A total of 242 patients (81% female) completed the questionnaire, with 137 patients from MCL and 105 from ZGT. The median age was 47 years [35–53], and the median BMI was 42 kg/m^2^ [39–47]. Hypertension and obstructive sleep apnea syndrome (OSAS) were the most prevalent obesity-related diseases. The highest level of completed education was vocational education (51%) (Table 1).

**Table 1:** Participant characteristics

	Total (*N* = 242)
Age, years ▪	47 [35–53]
Female ◦	195 (81)
Body weight, kg ▪	124 [113–140]
BMI, kg/m^2^ ▪	42 [39–47]
Baseline comorbidities ◦ - Hypertension - Diabetes mellitus - OSAS - Dyslipidemia - Osteoarthritis - GERD	74 (31) 27 (11) 63 (26) 28 (12) 57 (24) 39 (16)
Highest level of completed education ◦ - No degree - Primary school - High school—vocational education - High school – higher vocational education - Vocational education - Higher vocational education - University - Other	3 (1) 4 (2) 39 (16) 9 (4) 123 (51) 51 (21) 6 (3) 7 (3)

*BMI* body mass index, kg/m^2^, *OSAS* obstructive sleep apnea syndrome, *GERD* gastroesophageal reflux disease

The characteristics of the subgroups can be found in Table 2. Women exhibited a notably lower preoperative body weight than men (120 versus 143 kg; *p* < 0.001). In patients under 30 years old compared to those over 50 years old, preoperative body weight (133 versus 120 kg; *p* = 0.004) and BMI (45 versus 41 kg/m^2^; *p* < 0.001) were significantly higher. Patients with a BMI < 50 kg/m^2^ were notably older than those with a BMI ≥ 50 kg/m^2^ (49 versus 39 years; *p* = 0.017). Additionally, patients with obesity-related diseases were significantly older than those without obesity-related diseases (51 versus 34 years; *p* < 0.001), the proportion of males was higher in the group with obesity-related diseases (27% versus 7%; *p* < 0.001) (Table 2).

**Table 2:** Subgroup analyses for demographics, expected weight loss, and expected remission of comorbidities

	Gender			BMI, kg/m^2^			Age			Obesity-related diseases		
	*Men* *N* = 47	*Women* *N* = 195	*P* value	*BMI* < *50* *N* = 208	*BMI* ≥ *50* *N* = 33	*P* value	< *30 years* *N* = 41	> *50 years* *N* = 104	*P* value	*With obesity-related diseases* *N* = 153	*Without obesity-related diseases* *N* = 89	*P* value
*Demographics*												
Age, years ▪	50 [37–55]	47 [34–53]	0.248	49 [36–53]	39 [28–51]	**0.017**	25 [23–28]	54 [52–60]	** < 0.001**	51 [44–55]	34 [26–46]	** < 0.001**
Female ◦	-	195 (100)	-	170 (82)	24 (73)	0.225	35 (85)	79 (76)	0.214	112 (73)	83 (93)	** < 0.001**
Body weight, kg ▪	143 [132–169]	120 [110–131]*	** < 0.001**	120 [111–132]	165 [151—175]	** < 0.001**	133 [121–154]*	120 [110–137]	**0.004**	125 [113–139]	124 [113–140]*	0.772
BMI, kg/m^2^	42 [39–48]	42 [39–46]*	0.439	41 [39–44]	53 [51—56]	** < 0.001**	45 [41–50]*	41 [38–45]	** < 0.001**	42 [38–36]	42 [40–47]*	0.055
*Expected weight loss ▪*												
%TWL	30 [28–35]	32 [28–35]*	0.494	31 [28–35]*	35 [32—39]*	** < 0.001**	32 [29–39]*	32 [28–35]*	0.348	32 [28–35]*	33 [29–37]*	0.168
%EWL	78 [67–87]	81 [69–92]*	0.187	83 [72–92]*	68 [59 −76]*	** < 0.001**	76 [63–85]*	83 [69–92]*	**0.037**	82 [69—92]*	79 [68–89]*	0.650
*Expected remission of comorbidities* ◦/◦◦												
Hypertension	21 (45)	53 (27)	**0.019**	65 (31)	9 (27)	0.645	0	48 (46)	-	74 (48)	-	-
Total remission	14 (67)	31 (58)	0.066	41 (63)	4 (44)	0.552	-	29 (60)	-	45 (61)	-	-
Partial remission	7 (33)	17 (32)		19 (29)	5 (56)		-	16 (33)		24 (32)	-	
No remission	-	1 (2)		1 (2)	-		-	1 (2)		1 (1)	-	
Deterioration	-	-		-	-		-	-		-	-	
Missing	-	4 (8)		4 (6)	-		-	2 (4)		4 (5)	-	
Diabetes mellitus	8 (17)	19 (10)	0.155	22 (11)	5 (15)	0.439	0	17 (16)	-	27 (18)	-	-
Total remission	6 (75)	12 (63)	0.415	14 (64)	4 (80)	0.728	-	11 (65)	-	18 (67)	-	-
Partial remission	2 (25)	6 (32)		7 (32)	1 (20)		-	6 (35)		8 (30)	-	
No remission	-	-		-	-		-	-		-	-	
Deterioration	-	1 (5)		1 (5)	-		-	-		1 (4)	-	
Missing	-	-		-	-		-	-		-	-	
OSAS	28 (60)	35 (18)	** < 0.001**	49 (24)	14 (42)	**0.022**	4 (10)	37 (36)	**0.002**	63 (41)	-	-
Total remission	24 (86)	31 (89)	** < 0.001**	42 (86)	13 (93)	**0.005**	3 (75)	32 (86)	**0.008**	55 (87)	-	-
Partial remission	-	-		-	-		-	-		-	-	
No remission	3 (11)	2 (6)		5 (10)	-		1 (25)	4 (11)		5 (8)	-	
Deterioration	1 (4)	-		-	1 (7)		-	-		1 (2)	-	
Missing	-	2 (6)		2 (4)	-		-	1 (3)		2 (3)	-	
Dyslipidemia	10 (21)	18 (9)	**0.020**	24 (12)	4 (12)	0.923	0	20 (19)	-	28 (18)	-	-
Total remission	5 (50)	9 (50)	**0.022**	13 (54)	1 (25)	0.611	-	8 (40)	-	14 (50)	-	-
Partial remission	5 (50)	5 (28)		8 (33)	2 (50)		-	9 (45)		10 (36)	-	
No remission	-	3 (17)		2 (8)	1 (25)		-	2 (10)		3 (11)	-	
Deterioration	-	-		-	-		-	-		-	-	
Missing	-	1 (6)		1 (4)	-		-	1 (5)		1 (4)	-	
Osteoarthritis	8 (17)	49 (25)	0.240	55 (26)	2 (6)	**0.010**	0	46 (44)	-	57 (37)	-	-
Total remission	2 (25)	7 (14)	0.645	8 (15)	1 (50)	0.081	-	8 (17)	-	9 (16)	-	-
Partial remission	6 (75)	39 (80)		44 (80)	1 (50)		-	35 (76)		45 (79)	-	
No remission	-	1 (2)		1 (2)	-		-	1 (2)		1 (2)	-	
Deterioration	-	-		-	-		-	-		-	-	
Missing	-	2 (4)		2 (4)	-		-	2 (4)		2 (4)	-	
GERD	5 (11)	34 (17)	0.255	36 (17)	3 (9)	0.234	0	21 (20)	-	39 (25)	-	-
Total remission	2 (40)	16 (47)	0.699	18 (50)	-	**0.045**	-	6 (29)	-	18 (46)	-	-
Partial remission	1 (20)	11 (32)		11 (31)	1 (33)		-	10 (48)		12 (31)	-	
No remission	1 (20)	4 (12)		4 (11)	1 (33)		-	2 (10)		5 (13)	-	
Deterioration	-	1 (3)		-	1 (33)		-	1 (5)		1 (3)	-	
Missing	1 (20)	2 (6)		3 (8)	-		-	2 (10)		3 (8)	-	

▪Median [interquartile range]

◦Absolute number (percentage)

◦ Comorbidity percentages are calculated based on the prevalence within subgroups. Remission rates are calculated from the number of patients in each subgroup who have the specific comorbidity

*Number of missing values: 1 to 9

*BMI* body mass index, kg/m^2^, %*TWL* percentage total weight loss, *%EWL* percentage excess weight loss, *OSAS* obstructive sleep apnea syndrome, *GERD* gastroesophageal reflux disease

### The Top 3 Most Important Reasons for Undergoing MBS

Table 3 illustrates the primary reasons for opting for MBS, with mobility and health emerging as the predominant categories across all three top-ranked positions. Specifically, 21% of the patients mentioned mobility in the first place, 17% in the second place, followed by 18% in the third place. For the category of health, these percentages were 18%, 19%, and 12%, respectively. Additionally, pain reduction was ranked first by 19% of the participants, remission of obesity-related diseases was ranked second by 17%, and physical fitness was ranked third by 14% (Table 3).

**Table 3:** Top 3 most important reasons for undergoing MBS

	**Rank 1**	**Rank 2**	**Rank 3**
Children/family	4 (2)	8 (3)	12 (5)
Weight loss	21 (8)	6 (2)	18 (7)
Mobility	**57 (21)**	**46 (17)**	**46 (18)**
Physical fitness	31 (11)	45 (17)	**37 (14)**
Emotional well-being	1 (0)	2 (1)	13 (5)
Health	**50 (18)**	**50 (19)**	**31 (12)**
Fertility	1 (0)	3 (1)	-
Quality of life	5 (2)	1 (0)	5 (2)
Pain reduction	**51 (19)**	34 (13)	25 (10)
Participation	1 (0)	5 (2)	7 (3)
Remission of comorbidities	43 (16)	**46 (17)**	30 (12)
Follow-up surgery	4 (2)	1 (0)	-
Self-image	3 (1)	13 (5)	15 (6)
Other	3 (1)	4 (2)	6 (2)
Missing	-	2 (1)	16 (6)
Total	275 (100)	266 (100.0)	261 (100.0)

Data expressed as absolute frequencies and percentages (%)

### Expected Weight Loss

Patients reported an expected median %TWL of 32 [28–35] and a %EWL of 81 [69–90] (*N* = 241). Patients with BMI ≥ 50 kg/m^2^ expected more %TWL than patients with BMI < 50 kg/m^2^ (35% versus 31%; *p* < 0.001) (Table 2).

### Expected Remission of Obesity-related Diseases

The prevalence of obesity-related diseases and the expectations of the remission of obesity-related diseases are presented in Table 2 (columns obesity-related diseases). Most patients expected total remission of hypertension, diabetes mellitus, and OSAS. For dyslipidemia and gastroesophageal reflux disease (GERD), approximately half of the patients expected total remission. Partial remission is mainly expected for osteoarthritis (Table 2).

In subgroup 1 (gender), a higher prevalence of men with hypertension (45% versus 27%; *p* = 0.019), OSAS (60% versus 18%; *p* < 0.001), and dyslipidemia (21% versus 9%; *p* = 0.020) was observed. More women than men expected total remission of OSAS (89% versus 86%; *p* < 0.001). Conversely, more men than women reported higher expectations regarding the (partial) remission of dyslipidemia (total remission 50% versus 50%; partial remission 50% versus 28%; no remission 0% versus 17%; *p* = 0.022) (Table 2).

In subgroup 2 (BMI < 50 kg/m^2^ vs. BMI ≥ 50 kg/m^2^), a higher prevalence of OSAS was observed among patients with a BMI ≥ 50 kg/m^2^ (42% versus 24%; *p* = 0.022). These patients also expected higher remission rates of OSAS (93% versus 86%; *p* = 0.005). Conversely, patients with a BMI < 50 kg/m^2^ exhibited a higher prevalence of osteoarthritis (26% versus 6%; *p* = 0.010) and had higher expectations regarding GERD remission (total remission 50% versus 0%; partial remission 31% versus 33%; no remission 11% versus 33%; deterioration 0% versus 33%; *p* = 0.045) (Table 2).

In subgroup 3 (age < 30 years versus > 50 years), patients over 50 years had a higher prevalence of OSAS compared to patients under 30 years (36% versus 10%; *p* = 0.002) and also reported higher expectations of total remission (86% versus 75%; *p* = 0.008). No other obesity-related diseases were observed in patients under 30 years of age (Table 2).

### Expectations of Physical, Social, and Psychological Changes

Expectations of physical, social, and psychological changes after MBS are presented in Table 4. Given the open-ended nature of each domain, patients occasionally referenced categories that could be associated with another domain. Patients had the opportunity to provide multiple responses to a single question, resulting in a higher count of categories compared to the number of patients. Subsequently, these responses were categorized.

**Table 4:** Expected physical, social, and psychological changes after MBS

Physical (*N* = 625)		Social (*N* = 346)		Psychological (*N* = 338)	
*Overlap across all three domains*					
Weight loss	23 (4)	Weight loss	1 (0)	Weight loss	1 (0)
**Physical fitness**	**112 (18)**	Physical fitness	3 (1)	Physical fitness	6 (2)
Remission of comorbidities	38 (6)	Remission of comorbidities	1 (0)	Remission of comorbidities	1 (0)
**Mobility**	**162 (26)**	Mobility	4 (1)	Mobility	3 (1)
Appearance	107 (17)	Appearance	13 (4)	Appearance	10 (3)
Children/family	3 (1)	Children/family	1 (0)	Children/family	2 (1)
Emotional well-being	14 (2)	**Emotional well-being**	**50 (15)**	**Emotional well-being**	**81 (24)**
Participation	8 (1)	Participation	43 (12)	Participation	11 (3)
Self-image	7 (1)	**Self-image**	**75 (22)**	**Self-image**	**151 (45)**
*Overlap across two domains*					
		Interaction with others	25 (7)	Interaction with others	2 (1)
		Public spaces	24 (7)	Public spaces	2 (1)
		Reactions	31 (9)	Reactions	2 (1)
		**No change**	**54 (16)**	**No change**	**14 (4)**
**Pain reduction**	**122 (20)**			Pain reduction	3 (1)
Quality of life	3 (1)			Quality of life	1 (0)
Health	8 (1)			Health	4 (1)
*Unique categories for each domain*					
Sleep	1 (0)	Don’t know	13 (4)	Food and emotion	3 (1)
Fertility	1 (0)	Other	8 (2)	Balance	2 (1)
Follow-up surgery	1 (0)			Sleep	1 (0)
Other	15 (2)			Other	9 (3)

Data expressed as absolute frequencies and percentages (%)

Mobility, pain reduction, and physical fitness were the most frequently mentioned expected physical changes. In the context of social changes, self-image, no change, and emotional well-being emerged as the most frequently mentioned categories. Self-image and emotional well-being also arose as the first and second categories for expected psychological changes after surgery, followed by no expected changes in third place (Table 4).

Finally, other potential concerns after MBS were explored. In total, 77% of the participants expected no other problems (*N* = 195). Sixteen other potential concerns or topics were mentioned by 59 participants, among others “appearance” (*N* = 16), “complications” (*N* = 11), “anesthesia” (*N* = 3), “intake/nutrition” (*N* = 6), “rules for living” (*N* = 3), “quality of life” (*N* = 3), “medication” (*N* = 2), and “self-image” (*N* = 2).

### Impact of Complications

Table 5 presents the importance of being informed about the complication during the preoperative period, the anxiety of developing the complication, and the impact of the complication on the consideration of undergoing MBS. In general, patients considered it important to be informed about the complications (mean scores between 3.9 and 4.3). However, the complications did not induce anxiety (mean scores between 2.5 and 3.2) or heavily influence the consideration of undergoing MBS (mean scores between 2.6 and 3.0).

**Table 5:** Information, anxiety, and consideration about complications (*N* = 242*)

	**Information**	**Anxiety**	**Consideration**
Short-term complications (≤ 30 days after surgery)			
Bleeding	4.3 ± 0.8	2.7 ± 1.0	2.7 ± 1.1
Anastomotic leakage	4.3 ± 0.8	2.8 ± 1.0	2.7 ± 1.1
Wound infection	4.3 ± 0.8	2.6 ± 1.0	2.6 ± 1.1
Reoperation	4.2 ± 1.0	2.7 ± 1.0	2.8 ± 1.2
Pneumonia	4.0 ± 1.0	2.4 ± 1.0	2.6 ± 1.1
Thrombosis/pulmonary embolism	4.2 ± 1.0	2.7 ± 1.0	2.7 ± 1.1
Readmission	4.1 ± 1.0	2.6 ± 1.1	2.6 ± 1.2
Constipation	4.0 ± 1.0	2.5 ± 1.0	2.6 ± 1.1
Difficulties with eating and drinking/nausea and vomiting	4.3 ± 0.9	2.9 ± 1.1	2.8 ± 1.2
Long-term complications (> 2 years after surgery)			
Internal herniation	4.2 ± 1.0	3.2 ± 3.3	3.0 ± 1.1
Gastric ulcer	4.1 ± 1.0	2.6 ± 1.1	2.8 ± 1.1
GERD	4.1 ± 1.0	2.9 ± 1.0	2.8 ± 1.1
Dumping	4.2 ± 0.9	3.0 ± 1.1	2.9 ± 1.1
Malnutrition	4.1 ± 1.0	2.6 ± 1.0	2.7 ± 1.1
Underweight	4.0 ± 1.1	2.5 ± 1.0	2.7 ± 1.1
Less weight loss than expected	4.1 ± 1.0	3.1 ± 1.1	2.8 ± 1.1
Recurrent weight gain	4.2 ± 0.9	3.1 ± 1,1	2.9 ± 1.2
Reoperation	4.2 ± 1.0	2.6 ± 1.1	2.8 ± 1.2
Symptomatic cholelithiasis	3.9 ± 1.1	2.7 ± 1.1	2.7 ± 1.1
Complaints of hypoglycemia	4.0 ± 1.1	2.7 ± 1.0	2.7 ± 1.0
Diarrhea	3.9 ± 1.1	2.7 ± 1.0	2.8 ± 1.1
Deficiency in vitamins and/or minerals	4.2 ± 1.0	2.8 ± 1.1	2.9 ± 1.1

*Numbers of responses varying between 226 and 240 on each domain

Data expressed as mean ± standard deviation

Validation of the 5-point Likert scale: 1 = not important at all, 2 = not important, 3 = neutral, 4 = important, 5 = very important

*GERD* gastroesophageal reflux disease

Subgroup analyses on the impact of complications are shown in Appendix 2. In subgroup 1 (gender), women considered it significantly more important than men to be informed about anastomotic leakage, thrombosis/pulmonary embolism, constipation, difficulties with eating and drinking/nausea and vomiting, gastric ulcer, GERD, underweight, less weight loss than expected, reoperation (long-term), and symptomatic cholelithiasis. Women also reported greater anxiety about recurrent weight gain. No significant gender differences were observed in short- and long-term complications when considering surgery (Appendix 2, Table 6).

In subgroup 2 (BMI < 50 kg/m^2^ versus BMI ≥ 50 kg/m^2^), subgroup 3 (< 30 years versus > 50 years), and subgroup 4 (with versus without obesity-related diseases), no differences were observed in the reported importance of being informed about the complications, anxiety related to potential complications, or the impact of complications on the decision to undergo MBS (Appendix 2, Tables 7, 8, and 9).

## Discussion

This study aimed to explore patients’ main expected concerns, barriers, and challenges in the long term after MBS, in conjunction with patients’ preoperative expectations regarding physical, social, and psychological aspects. Despite prioritizing being informed about potential complications, it did not discourage patients from choosing MBS.

Mobility, health, and remission of obesity-related diseases emerged as the most important reasons for undergoing surgery. This is in line with previous literature showing that the primary motivation for individuals pursuing MBS is connected to physical health [16–20]. While weight loss has also been recognized as an important motivation in previous literature [20, 21], it did not emerge as one of the three primary reasons in the current study. Interestingly, Hult et al. identified weight loss as the leading motivation, not only in their overall study population but also in a subgroup of patients from the Netherlands [20]. An explanation for this difference might be the higher observed BMI in their population, compared to a lower BMI in the current study, suggesting that individuals with a higher BMI may prioritize weight loss as a more important reason for opting for MBS. This hypothesis may align with our findings of a higher expected %TWL in patients with a BMI ≥ 50 kg/m^2^ compared to patients with BMI < 50 kg/m^2^ (35% versus 31%; *p* < 0.001). Another explanation for why weight loss did not emerge as the primary reason for seeking MBS could be the emphasis placed during the preoperative information session on obesity as a metabolic disease, rather than merely a result of lifestyle choices or excess weight. This may have shifted the participants’ focus beyond weight loss alone.

Although weight loss was not frequently mentioned as the most important reason for undergoing MBS, participants in this study expressed high expectations for postoperative weight loss, with a median %TWL of 32% and a %EWL of 81%. This aligns with previous studies, reporting expected %EWL ranging from 71 to 94% [16, 20, 21]. However, gastric bypass surgery has been associated with reported %EWL ranging from 27 to 69% after 10 years [23]. This shows a considerable disparity between anticipated and realized weight loss outcomes, which can result in postoperative disappointment. Nowadays, it is preferable to quantify weight loss in terms of %TWL rather than %EWL [27]. Unfortunately, previous studies relied solely on %EWL for their measurements, making a reliable comparison between their results and the outcomes of this study infeasible. In the questionnaire, no specific timeframe for expected weight loss after MBS was defined. Consequently, participants might have referred to expected weight loss in the short term after surgery rather than in the long term.

This study reveals a notable difference in weight loss expectations between age groups, with patients over 50 years anticipating more excess weight loss compared to those under 30 years (83% versus 76%; *p* = 0.037). However, %TWL did not differ between the age groups. These findings contradict previous studies indicating that younger patients tend to have higher expectations regarding weight loss [28, 29]. A potential explanation for the difference in weight loss expectations could be that older patients believe they need to lose more weight to improve their obesity-related diseases, which were more prevalent in the older group. In this study, OSAS was the only obesity-related disease observed in the < 30 age group, whereas all other obesity-related diseases were present in the > 50 age group.

Although participants had unrealistic weight loss expectations, their estimates of obesity-related disease resolution were accurate. The anticipated total remission rates for obesity-related diseases were as follows: 61% for hypertension, 87% for OSAS, and 67% for diabetes mellitus. These rates align closely with those reported in a meta-analysis by Buchwald et al., where hypertension resolution occurred in 62% of the patients and OSAS resolution in 86% [30]. However, the expected total remission rate of diabetes mellitus in our population was lower than the actual remission rate reported by Buchwald et al. (67% vs. 77%) [30]. Similarly to our expected remission rate, Svanenik et al. reported an actual diabetes remission rate of 67% in their patients [31]. Overall, these findings suggest that our preoperative information session on MBS is effective regarding obesity-related diseases. Patients older than 50 years anticipated higher resolution rates for OSAS compared to their younger counterparts. Notably, these higher expected remission rates align with those reported in the literature, suggesting that older patients’ expectations are realistic [30]. Unfortunately, it was not possible to compare the expected remission rates for other obesity-related diseases, as patients younger than 30 years did not have any additional obesity-related diseases. In the literature, the reported remission rate of obesity-related diseases is higher in younger adults compared to older individuals [32].

Our findings suggest a prioritization of physical factors over social and psychological factors when seeking MBS. Although the expected changes in physical, social, and psychological domains after MBS were addressed separately through open-ended questions, there was overlap in the responses across these domains. Differentiating between the domains appears challenging for patients, which is understandable given the interconnected nature of these domains. For instance, feeling better when in the company of others can be categorized as both psychological and social. Mobility, pain reduction, and physical fitness emerged as primary motivations for individuals considering MBS in our study, alongside considerations such as improved health and remission of obesity-related diseases. Consistent with prior research, low percentages of self-image and emotional well-being were seen as the top three motivations for undergoing MBS (33). Conversely, findings from a study conducted by Hult et al. revealed that self-esteem, mental health, and social life were ranked as very important by 72%, 76%, and 56% of the participants, respectively [14, 20]. However, their study considered a broad spectrum of motivations to undergo MBS as very important.

Our results show that patients consider it important to be informed about both short-term and long-term complications. However, the complications do not induce anxiety or heavily influence the consideration of undergoing MBS. This aligns with the conclusions drawn by van Rijswijk et al., who found that patients are willing to accept considerable risks, including short-term complications (≤ 30 days after surgery), acute internal herniation (≥ 30 days), and mortality (≤ 30 days), to achieve their weight loss goals [25]. However, only short-term complications were incorporated in the study of van Rijswijk et al. [25], while this study also included long-term complications. Furthermore, this study conducted subgroup analyses to assess patients’ perceptions of complications. The primary findings from these analyses indicate that female patients expressed a greater desire for comprehensive information and reported higher levels of anxiety about recurrent weight gain. These subgroup differences should be considered when discussing treatment options with patients, such as taking more time for explanations or referring them to reliable sources.

## Strengths and Limitations

The strengths of this study are the large sample size and the administration of the questionnaire in two independent hospitals. Additionally, it expands upon previous literature by adding patients’ main expected concerns, barriers, or challenges in the long term after MBS. Our study has some limitations that need to be mentioned: first, the questionnaire is not validated. However, it was developed based on literature and clinical expertise. Second, the questionnaire did not inquire about the preferred MBS procedure, while this could influence results regarding weight loss expectations or specific complications. Third, the generalizability of the study may be limited as this study was solely conducted in the Netherlands. Administering the questionnaire globally could enhance generalizability. Finally, due to the study design, it was not possible to compare preoperative expectations with postoperative outcomes. Nevertheless, to answer our research question, the cross-sectional study design was deemed appropriate. Future studies could explore the concordance between patients’ preoperative expectations and postoperative outcomes.

## Conclusion

In conclusion, improvement of mobility and health were the main reasons for undergoing MBS. Although participants had high weight loss expectations, their estimates of obesity-related disease resolution were accurate. Despite prioritizing being informed information about potential complications, it did not discourage patients from choosing MBS. While the study was conducted in two Dutch centers, the insights from this study are crucial for clinicians worldwide as they highlight the importance of addressing patient expectations and providing thorough information on potential outcomes and complications to ensure informed decision-making. Preoperative discussions about expectations are crucial in this regard to prevent potential disappointment.

## Supplementary Information

Below is the link to the electronic supplementary material.

Supplementary file1 (DOCX 211 KB)

## Data Availability

Data is provided within the manuscript or supplementary information files. The database is available upon reasonable request.
