# Supplementary material for: Patient Expectations of Bariatric Outcomes, Baseline, and Long-term Evaluation
Source: Obes Surg. 2025 Jul 4;35(8):3064–74. doi: 10.1007/s11695-025-07997-0 (PMC12380636; doi:10.1007/s11695-025-07997-0)
Supplement: Supplementary file 1 — Supplementary file1 (DOCX 211 KB) [file 11695_2025_7997_MOESM1_ESM.docx]

# Appendix 1

Dear sir/madam,

We kindly ask you to participate in a scientific research study conducted by the Medical Centre Leeuwarden (MCL) in collaboration with Hospital Group Twente (ZGT) Almelo/Hengelo. You are invited to participate in this study, because you are on the waiting list for metabolic bariatric surgery (MBS).

There can be various reasons for undergoing MBS. In order to provide the patient with the correct information prior to the operation, it is important to gain insight into the patient's motivation for undergoing MBS and their expectations of this procedure. This study aims to gain insight into these aspects. We would like to ask you to participate in this study by completing the questionnaire below. It will take approximately 10 minutes to complete.

By completing the questionnaire, you agree that:

- Your answers will be included anonymously in the study;
- The anonymous data will be exchanged between MCL and ZGT Almelo/Hengelo, as this study is a collaboration between these two hospitals;
- The anonymous data will be stored for a period of 15 years;
- The anonymous data will be used for writing and publishing a scientific article.

On the next page, you will find the questionnaire.

1. What is your gender? Please circle your answer.
2. Male
3. Female
4. Other
5. What is your age

……………………………………………………………………………………years

1. What is your current weight in kilograms?

……………………………………………………………………………… kilograms

1. What is your length in meters?

……………………………………………………………………………………meters

1. What is the highest level of education you have completed? Please circle your answer.
   1. I have not completed any education
   2. Primary school
   3. High school - vocational education (VMBO)
   4. High school - higher vocational education (HAVO)
   5. High school - pre-university education (VWO/Gymnasium)
   6. Vocational education (MBO)
   7. Higher vocational education (HBO)
   8. University
   9. I have completed another type of education, namely ...
2. What is the reason you want to undergo MBS? Please state the 3 most important reasons (you can think of reasons such as: reducing medication use, increased mobility, reduced pain, increased energy, etc.)
   1. ………………………………………………..…………………………………
   2. ………………………………………………..…………………………………
   3. ………………………………………………..………………………………
3. How many kilograms do you expect to lose after surgery?

……………………………………………………………………………… kilograms

1. Do you have any of the following health problems? You can select multiple answers. Please circle the answers that apply to you:
   1. Sleep apnea (breathing cessation during sleep), for which you use a brace or mask
   2. High blood pressure, for which you take medication
   3. Diabetes mellitus, for which you take medication
   4. High cholesterol, for which you take medication
   5. Osteoarthritis, diagnosed by X-ray or scan
   6. Complaints of reflux, for which you take medication

**If you do not have any of the mentioned health problems in question 8, please proceed to question 10.**

1. What do you expect will happen to the above-mentioned health problems after the surgery? Please circle your answer (only fill in for the answers you circled in question 8).
   1. Sleep apnea (breathing cessation during sleep), for which you use a brace or mask
   2. Brace or mask not needed anymore
   3. Continued use of the brace or mask
   4. More support needed from the mask
2. High blood pressure, for which you take medication
3. Improvement (no medication needed anymore)
4. Improvement (less medication needed)
5. No change in medication
6. More medication needed
7. Diabetes mellitus, for which you take medication
   1. Improvement (no medication needed anymore)
   2. Improvement (less medication needed)
   3. No change in medication
   4. More medication needed
8. High cholesterol, for which you take medication
   1. Improvement (no medication needed anymore)
   2. Improvement (less medication needed)
   3. No change in medication
   4. More medication needed
9. Osteoarthritis, diagnosed by X-ray or scan
   1. Improvement (no pain complaints anymore)
   2. Improvement (less pain complaints)
   3. No change in pain complaints
   4. More pain complaints
10. Complaints of reflux, for which you take medication.
    1. Improvement (no medication needed anymore)
    2. Improvement (less medication needed)
    3. No change in medication
    4. More medication needed
11. What changes do you expect to occur physically after the surgery? Consider things like reduced pain, increased mobility, excess skin, etc.

……………………………………………………………………………………………………………………………………………………………………………………

1. What changes do you expect to occur socially after the surgery? Consider potential changes in interactions with other people, how you feel in public spaces, etc.

……………………………………………………………………………………………………………………………………………………………………………………

1. What changes do you expect to occur psychologically after the surgery? Consider things like self-image, emotional well-being, etc.

……………………………………………………………………………………………………………………………………………………………………………………

1. How do you view the potential problems that can occur during and within the first 30 days after the surgery? Mark the importance of each problem in the following categories:
   1. **Information**: How important is it for you to be informed about the mentioned problems prior to the surgery?

1 = not important at all, 2 = not important, 3 = neutral, 4 = important, 5 = very important

- 1. **Anxiety:** How anxious are you about experiencing the mentioned problems around the time of the surgery and within the first 30 days after the surgery?

1 = not anxious at all, 2 = not anxious, 3 = neutral, 4 = anxious, 5 = very anxious

- 1. **Consideration**: How much do the following problems weigh in your decision to undergo or not undergo the surgery?

1 = not important at all, 2 = not important, 3 = neutral, 4 = important, 5 = very important

|  | **Information** | | | | | **Anxiety** | | | | | **Consideration** | | | | |
| --- | --- | --- | --- | --- | --- | --- | --- | --- | --- | --- | --- | --- | --- | --- | --- |
|  | 1 | 2 | 3 | 4 | 5 | 1 | 2 | 3 | 4 | 5 | 1 | 2 | 3 | 4 | 5 |
| Bleeding |  |  |  |  |  |  |  |  |  |  |  |  |  |  |  |
| Anastomotic leakage |  |  |  |  |  |  |  |  |  |  |  |  |  |  |  |
| Wound infection |  |  |  |  |  |  |  |  |  |  |  |  |  |  |  |
| Reoperation |  |  |  |  |  |  |  |  |  |  |  |  |  |  |  |
| Pneumonia |  |  |  |  |  |  |  |  |  |  |  |  |  |  |  |
| Thrombosis/ pulmonary embolism |  |  |  |  |  |  |  |  |  |  |  |  |  |  |  |
| Readmission |  |  |  |  |  |  |  |  |  |  |  |  |  |  |  |
| Constipation |  |  |  |  |  |  |  |  |  |  |  |  |  |  |  |
| Difficulty with eating and drinking/ nausea and vomiting |  |  |  |  |  |  |  |  |  |  |  |  |  |  |  |

1. How do you view the potential problems that can occur in the long-term (>2 years) after the surgery? Mark the importance of each problem in the following categories:
   1. **Information**: How important is it for you to be informed about the mentioned problems prior to the surgery?

1 = not important at all, 2 = not important, 3 = neutral, 4 = important, 5 = very important

- 1. **Anxiety:** How anxious are you about experiencing the mentioned problems in the long term (more than 2 years) after the surgery?

1 = not anxious at all, 2 = not anxious, 3 = neutral, 4 = anxious, 5 = very anxious

- 1. **Consideration**: How much do the following problems weigh in your decision to undergo or not undergo the surgery?

1 = not important at all, 2 = not important, 3 = neutral, 4 = important, 5 = very important

|  | **Information** | | | | | **Anxiety** | | | | | **Consideration** | | | | |
| --- | --- | --- | --- | --- | --- | --- | --- | --- | --- | --- | --- | --- | --- | --- | --- |
|  | 1 | 2 | 3 | 4 | 5 | 1 | 2 | 3 | 4 | 5 | 1 | 2 | 3 | 4 | 5 |
| Internal herniation |  |  |  |  |  |  |  |  |  |  |  |  |  |  |  |
| Gastric ulcer |  |  |  |  |  |  |  |  |  |  |  |  |  |  |  |
| Reflux |  |  |  |  |  |  |  |  |  |  |  |  |  |  |  |
| Dumping |  |  |  |  |  |  |  |  |  |  |  |  |  |  |  |
| Malnutrition |  |  |  |  |  |  |  |  |  |  |  |  |  |  |  |
| Underweight |  |  |  |  |  |  |  |  |  |  |  |  |  |  |  |
| Less weight loss than expected |  |  |  |  |  |  |  |  |  |  |  |  |  |  |  |
| Recurrent weight gain |  |  |  |  |  |  |  |  |  |  |  |  |  |  |  |
|  | **Information** | | | | | **Anxiety** | | | | | **Consideration** | | | | |
|  | 1 | 2 | 3 | 4 | 5 | 1 | 2 | 3 | 4 | 5 | 1 | 2 | 3 | 4 | 5 |
| Reoperation |  |  |  |  |  |  |  |  |  |  |  |  |  |  |  |
| Symptomatic cholelithiasis |  |  |  |  |  |  |  |  |  |  |  |  |  |  |  |
| Complaints of hypoglycemia |  |  |  |  |  |  |  |  |  |  |  |  |  |  |  |
| Diarrhea |  |  |  |  |  |  |  |  |  |  |  |  |  |  |  |
| Deficiency in vitamins and/or minerals |  |  |  |  |  |  |  |  |  |  |  |  |  |  |  |

1. Are there any other potential problems you fear after the surgery that were not addressed in the questions above?

……………………………………………………………………………………………………………………………………………………………………………………

This was the last question of the questionnaire. You can hand in the completed questionnaire at the reception desk. You can also return the form using the enclosed envelope. No stamp is required. Thank you for completing the questionnaire, and we wish you success with the surgery and recovery afterward!

# Appendix 2

| **Table 6 –** Impact of complications, subgroup analysis based on gender | | | | | | | | | |
| --- | --- | --- | --- | --- | --- | --- | --- | --- | --- |
|  | **Information** | | | **Anxiety** | | | **Consideration** | | |
|  | **Men**  N=47* | **Women**  N=195* | P value^×^ | **Men**  N=47* | **Women**  N=195* | P value^×^ | **Men**  N=47* | **Women**  N=195* | P value^×^ |
| **Short-term complications (≤ 30 days after surgery)** | | | | | | | | | |
| Bleeding | 4.1 ± 1.0 | 4.4 ± 0.8 | 0.185 | 2.5 ± 1.1 | 2.8 ± 1.0 | 0.452 | 2.4 ± 1.1 | 2.8 ± 1.1 | 0.247 |
| Anastomotic leakage | 4.0 ± 0.9 | 4.4 ± 0.8 | **0.044** | 2.4 ± 1.0 | 2.8 ± 1.0 | 0.122 | 2.4 ± 1.1 | 2.8 ± 1.1 | 0.264 |
| Wound infection | 4.0 ± 0.9 | 4.3 ± 0.8 | 0.069 | 2.5 ± 1.0 | 2.7 ± 1.0 | 0.586 | 2.5 ± 1.2 | 2.7 ± 1.1 | 0.689 |
| Reoperation | 3.7 ± 1.0 | 4.3 ± 0.9 | 0.264 | 2.3 ± 0.9 | 2.7 ± 1.1 | 0.176 | 2.4 ± 1.2 | 2.8 ± 1.1 | 0.152 |
| Pneumonia | 3.5 ± 1.1 | 4.2 ± 1.0 | 0.132 | 2.3 ± 1.1 | 2.5 ± 1.0 | 0.709 | 2.2 ± 1.2 | 2.6 ± 1.1 | 0.187 |
| Thrombosis/ pulmonary embolism | 3.7 ± 1.2 | 4.3 ± 0.9 | **0.041** | 2.5 ± 1.1 | 2.7 ± 1.0 | 0.545 | 2.4 ± 1.2 | 2.8 ± 1.1 | 0.259 |
| Readmission | 3.5 ± 1.1 | 4.2 ± 0.9 | 0.088 | 2.3 ± 1.0 | 2.7 ± 1.1 | 0.332 | 2.3 ± 1.0 | 2.7 ± 1.2 | 0.116 |
| Constipation | 3.5 ± 1.2 | 4.1 ± 0.9 | **0.038** | 2.4 ± 1.0 | 2.6 ± 1.0 | 0.552 | 2.3 ± 1.2 | 2.6 ± 1.1 | 0.344 |
| Difficulties with eating and drinking/ nausea and vomiting | 3.9 ± 1.1 | 4.4 ± 0.8 | **0.038** | 2.5 ± 1.1 | 3.0 ± 1.1 | 0.066 | 2.5 ± 1.2 | 2.9 ± 1.2 | 0.182 |
| **Long-term complications** | | | | | | | | | |
| Internal herniation | 3.7 ± 1.1 | 4.4 ± 0.9 | 0.066 | 2.6 ± 1.1 | 3.1 ± 1.0 | 0.062 | 2.8 ± 1.3 | 3.0 ± 1.1 | 0.574 |
| Gastric ulcer | 3.6 ± 1.1 | 4.2 ± 0.9 | **0.033** | 2.5 ± 1.0 | 2.8 ± 0.9 | 0.333 | 2.8 ± 1.2 | 2.8 ± 1.0 | 0.987 |
| GERD | 3.6 ± 1.2 | 4.2 ± 0.9 | **0.029** | 2.5 ± 0.9 | 3.0 ± 1.0 | 0.157 | 2.6 ± 1.1 | 2.8 ± 1.0 | 0.435 |
| Dumping | 3.9 ± 1.1 | 4.3 ± 0.8 | 0.141 | 2.7 ± 1.1 | 3.1 ± 1.0 | 0.103 | 2.8 ± 1.2 | 3.0 ± 1.1 | 0.815 |
| Malnutrition | 3.8 ± 1.1 | 4.1 ± 1.0 | 0.248 | 2.5 ± 1.0 | 2.6 ± 1.0 | 0.891 | 2.8 ± 1.2 | 2.7 ± 1.1 | 0.767 |
| Underweight | 3.6 ± 1.1 | 4.1 ± 1.0 | **0.035** | 2.4 ± 0.9 | 2.5 ± 1.0 | 0.770 | 2.7 ± 1.0 | 2.6 ± 1.1 | 0.893 |
| Less weight loss than expected | 3.7 ± 1.1 | 4.2 ± 0.9 | **0.026** | 2.7 ± 1.1 | 3.2 ± 1.0 | 0.145 | 2.8 ± 1.2 | 2.9 ± 1.1 | 0.897 |
| Recurrent weight gain | 3.9 ± 1.1 | 4.3 ± 0.9 | 0.084 | 2.6 ± 1.1 | 3.2 ± 1.1 | **0.033** | 2.8 ± 1.3 | 2.9 ± 1.2 | 0.838 |
| Reoperation | 3.6 ± 1.2 | 4.1 ± 1.0 | **0.024** | 2.4 ± 1.0 | 2.7 ± 1.0 | 0.248 | 2.8 ± 1.1 | 2.9 ± 1.0 | 0.816 |
| Symptomatic cholelithiasis | 3.4 ± 1.3 | 4.1 ± 1.0 | **0.022** | 2.4 ± 1.0 | 2.8 ± 1.0 | 0.246 | 2.4 ± 1.1 | 2.8 ± 1.1 | 0.332 |
| Complaints of hypoglycemia | 3.5 ± 1.2 | 4.1 ± 1.0 | 0.053 | 2.5 ± 1.0 | 2.7 ± 1.0 | 0.589 | 2.7 ± 1.0 | 2.8 ± 1.0 | 0.917 |
| Diarrhea | 3.6 ± 1.2 | 4.0 ± 1.1 | 0.320 | 2.6 ± 1.1 | 2.7 ± 1.0 | 0.860 | 2.7 ± 1.1 | 2.8 ± 1.0 | 0.753 |
| Deficiency in vitamins and/or minerals | 3.9 ± 1.2 | 4.3 ± 0.9 | 0.147 | 2.6 ± 1.0 | 2.9 ± 1.1 | 0.259 | 2.8 ± 1.1 | 2.9 ± 1.1 | 0.770 |
| *Numbers of responses varying between 43 – 47 (men) and 181 - 194 (women) on each domain.  ^×^Adjusted P value (Benjamini-Hochberg correction).  Data expressed as mean ± standard deviation. Validation of the 5-point Likert scale: 1 = not important at all, 2 = not important, 3 = neutral, 4 = important, 5 = very important. GERD = gastroesophageal reflux disease. | | | | | | | | | |

| **Table 7 –** Impact of complications, subgroup analysis based on BMI | | | | | | | | | |
| --- | --- | --- | --- | --- | --- | --- | --- | --- | --- |
|  | **Information** | | | **Anxiety** | | | **Consideration** | | |
|  | **BMI < 50 kg/m^2^**  N=208* | **BMI ≥ 50 kg/m^2^**  N=33* | P value^×^ | **BMI < 50 kg/m^2^**  N=208* | **BMI ≥ 50 kg/m^2^**  N=33* | P value^×^ | **BMI < 50 kg/m^2^**  N=208* | **BMI ≥ 50 kg/m^2^**  N=33* | P value^×^ |
| **Short-term complications (≤ 30 days after surgery)** | | | | | | | | | |
| Bleeding | 4.4 ± 0.8 | 4.1 ± 1.2 | 0.894 | 2.7 ± 1.0 | 2.7 ± 1.0 | 0.968 | 2.8 ± 1.1 | 2.3 ± 1.1 | 0.241 |
| Anastomotic leakage | 4.3 ± 0.8 | 4.2 ± 1.1 | 0.943 | 2.8 ± 1.0 | 2.8 ± 1.0 | 0.977 | 2.8 ± 1.1 | 2.1 ± 1.1 | 0.059 |
| Wound infection | 4.3 ± 0.8 | 4.3 ± 1.0 | 0.989 | 2.6 ± 1.0 | 2.7 ± 1.0 | 0.890 | 2.7 ± 1.1 | 2.2 ± 1.1 | 0.190 |
| Reoperation | 4.1 ± 1.0 | 4.3 ± 1.0 | 0.811 | 2.6 ± 1.1 | 2.8 ± 0.9 | 0.415 | 2.8 ± 1.2 | 2.4 ± 1.1 | 0.259 |
| Pneumonia | 4.0 ± 1.1 | 4.3 ± 1.0 | 0.471 | 2.4 ± 1.0 | 2.6 ± 1.1 | 0.521 | 2.6 ± 1.1 | 2.3 ± 1.1 | 0.462 |
| Thrombosis/ pulmonary embolism | 4.2 ± 1.0 | 4.1 ± 1.2 | 0.985 | 2.7 ± 1.0 | 2.9 ± 1.0 | 0.407 | 2.8 ± 1.1 | 2.2 ± 1.0 | 0.166 |
| Readmission | 4.1 ± 1.0 | 4.0 ± 1.2 | 0.985 | 2.6 ± 1.1 | 2.7 ± 1.0 | 0.859 | 2.7 ± 1.1 | 2.4 ± 1.2 | 0.433 |
| Constipation | 4.0 ± 1.0 | 3.9 ± 1.2 | 0.971 | 2.5 ± 1.0 | 2.5 ± 1.0 | 0.992 | 2.6 ± 1.2 | 2.2 ± 1.0 | 0.337 |
| Difficulties with eating and drinking/ nausea and vomiting | 4.3 ± 0.9 | 4.1 ± 1.2 | 0.939 | 2.9 ± 1.1 | 3.2 ± 1.1 | 0.415 | 2.9 ± 1.2 | 2.5 ± 1.1 | 0.330 |
| **Long-term complications (> 2 years after surgery)** | | | | | | | | | |
| Internal herniation | 4.3 ± 0.9 | 4.0 ± 1.1 | 0.518 | 3.0 ± 1.0 | 3.0 ± 1.0 | 0.906 | 3.0 ± 1.1 | 2.7 ± 1.1 | 0.459 |
| Gastric ulcer | 4.1 ± 1.0 | 3.9 ± 1.2 | 0.807 | 2.8 ± 0.9 | 2.8 ± 1.0 | 0.986 | 2.8 ± 1.0 | 2.5 ± 1.1 | 0.454 |
| GERD | 4.1 ± 0.9 | 3.8 ± 1.2 | 0.586 | 2.9 ± 1.0 | 2.8 ± 1.1 | 1.0 | 2.8 ± 1.0 | 2.5 ± 1.1 | 0.466 |
| Dumping | 4.3 ± 0.9 | 4.0 ± 1.1 | 0.558 | 3.0 ± 1.1 | 2.9 ± 1.0 | 0.892 | 3.0 ± 1.1 | 2.6 ± 1.2 | 0.356 |
| Malnutrition | 4.1 ± 1.0 | 3.7 ± 1.3 | 0.248 | 2.6 ± 1.0 | 2.5 ± 0.9 | 0.847 | 2.8 ± 1.1 | 2.4 ± 1.0 | 0.423 |
| Underweight | 4.1 ± 1.0 | 3.5 ± 1.3 | 0.155 | 2.5 ± 1.0 | 2.4 ± 1.0 | 0.807 | 2.7 ± 1.1 | 2.4 ± 1.0 | 0.472 |
| Less weight loss than expected | 4.1 ± 1.0 | 4.2 ± 0.8 | 1.0 | 3.1 ± 1.1 | 3.2 ± 1.0 | 0.661 | 2.9 ± 1.1 | 2.8 ± 1.1 | 0.937 |
| Recurrent weight gain | 4.2 ± 0.9 | 4.1 ± 1.0 | 0.637 | 3.1 ± 1.1 | 3.1 ± 1.2 | 0.909 | 2.9 ± 1.2 | 2.7 ± 1.2 | 0.678 |
| Reoperation | 4.1 ± 1.0 | 3.8 ± 1.2 | 0.660 | 2.7 ± 1.0 | 2.7 ± 1.0 | 0.937 | 2.9 ± 1.0 | 2.7 ± 1.1 | 0.611 |
| Symptomatic cholelithiasis | 4.0 ± 1.1 | 3.6 ± 1.2 | 0.331 | 2.7 ± 1.1 | 2.4 ± 1.2 | 0.516 | 2.7 ± 1.1 | 2.4 ± 1.1 | 0.428 |
| Complaints of hypoglycemia | 4.0 ± 1.1 | 3.8 ± 1.2 | 0.638 | 2.7 ± 1.0 | 2.5 ± 1.0 | 0.632 | 2.8 ± 1.0 | 2.3 ± 1.0 | 0.178 |
| Diarrhea | 4.0 ± 1.1 | 3.7 ± 1.2 | 0.435 | 2.8 ± 1.0 | 2.5 ± 1.1 | 0.631 | 2.8 ± 1.0 | 2.4 ± 1.2 | 0.274 |
| Deficiency in vitamins and/or minerals | 4.3 ± 0.9 | 3.9 ± 1.2 | 0.332 | 2.9 ± 1.0 | 2.6 ± 1.1 | 0.466 | 2.9 ± 1.1 | 2.5 ± 1.2 | 0.174 |
| *Numbers of responses varying between 195 - 207 (BMI < 50 kg/m^2^) and 30 - 33 (BMI ≥ 50 kg/m^2^) on each domain. ^×^Adjusted P value (Benjamini-Hochberg correction).  Data expressed as mean ± standard deviation. Validation of the 5-point Likert scale: 1 = not important at all, 2 = not important, 3 = neutral, 4 = important, 5 = very important. GERD = gastroesophageal reflux disease. | | | | | | | | | |

| **Table 8 –** Impact of complications, subgroup analysis based on age | | | | | | | | | |
| --- | --- | --- | --- | --- | --- | --- | --- | --- | --- |
|  | **Information** | | | **Anxiety** | | | **Consideration** | | |
|  | **< 30 years**  N=41* | **>50 years**  N=104* | P value^×^ | **< 30 years**  N=41* | **>50 years**  N=104* | P value^×^ | **< 30 years**  N=41* | **>50 years**  N=104* | P value^×^ |
| **Short-term complications (≤ 30 days after surgery)** | | | | | | | | | |
| Bleeding | 4.4 ± 0.6 | 4.4 ± 0.8 | 1.0 | 2.9 ± 0.9 | 2.7 ± 1.0 | 0.627 | 2.6 ± 1.1 | 2.7 ± 1.0 | 0.893 |
| Anastomotic leakage | 4.4 ± 0.7 | 4.3 ± 0.8 | 0.892 | 2.9 ± 1.0 | 2.8 ± 1.0 | 0.819 | 2.6 ± 1.2 | 2.8 ± 1.0 | 0.644 |
| Wound infection | 4.4 ± 0.7 | 4.3 ± 0.8 | 1.0 | 2.6 ± 0.9 | 2.7 ± 1.0 | 0.997 | 2.4 ± 1.2 | 2.7 ± 1.0 | 0.273 |
| Reoperation | 4.4 ± 0.7 | 4.1 ± 1.0 | 0.503 | 2.7 ± 1.1 | 2.6 ± 1.1 | 0.787 | 2.7 ± 1.2 | 2.7 ± 1.1 | 0.991 |
| Pneumonia | 4.2 ± 0.9 | 4.0 ± 1.1 | 0.891 | 2.3 ± 0.8 | 2.6 ± 1.0 | 0.373 | 2.4 ± 1.1 | 2.6 ± 1.1 | 0.498 |
| Thrombosis/ pulmonary embolism | 4.4 ± 0.8 | 4.2 ± 1.0 | 0.759 | 2.6 ± 1.0 | 2.7 ± 1.0 | 0.850 | 2.6 ± 1.1 | 2.7 ± 1.1 | 0.772 |
| Readmission | 4.3 ± 0.9 | 4.0 ± 1.1 | 0.662 | 2.6 ± 1.2 | 2.6 ± 1.0 | 0.905 | 2.5 ± 1.2 | 2.7 ± 1.1 | 0.721 |
| Constipation | 4.2 ± 0.9 | 4.0 ± 1.0 | 0.711 | 2.4 ± 1.2 | 2.6 ± 0.9 | 0.518 | 2.5 ± 1.2 | 2.7 ± 1.1 | 0.468 |
| Difficulties with eating and drinking/ nausea and vomiting | 4.3 ± 0.9 | 4.3 ± 0.9 | 0.974 | 3.2 ± 1.0 | 2.8 ± 1.0 | 0.169 | 2.8 ± 1.2 | 2.8 ± 1.1 | 0.982 |
| **Long-term complications (> 2 years after surgery)** | | | | | | | | | |
| Internal herniation | 4.5 ± 0.7 | 4.1 ± 1.0 | 0.326 | 3.2 ± 0.9 | 2.8 ± 1.0 | 0.331 | 3.1 ± 1.2 | 2.9 ± 1.0 | 0.878 |
| Gastric ulcer | 4.3 ± 0.7 | 4.0 ± 1.0 | 0.565 | 2.8 ± 1.0 | 2.7 ± 0.9 | 0.852 | 2.6 ± 1.1 | 2.8 ± 0.9 | 0.432 |
| GERD | 4.1 ± 0.8 | 4.1 ± 0.9 | 0.995 | 2.7 ± 0.9 | 2.9 ± 1.0 | 0.370 | 2.6 ± 1.1 | 2.8 ± 0.9 | 0.331 |
| Dumping | 4.4 ± 0.8 | 4.2 ± 0.9 | 0.719 | 3.0 ± 1.1 | 3.0 ± 1.1 | 0.935 | 2.9 ± 1.3 | 2.9 ± 1.0 | 0.934 |
| Malnutrition | 4.2 ± 1.0 | 4.0 ± 1.0 | 0.759 | 2.4 ± 1.0 | 2.7 ± 1.0 | 0.375 | 2.4 ± 1.2 | 2.8 ± 1.0 | 0.242 |
| Underweight | 4.2 ± 1.0 | 4.0 ± 1.0 | 0.718 | 2.3 ± 0.9 | 2.6 ± 1.0 | 0.368 | 2.4 ± 1.1 | 2.7 ± 1.0 | 0.350 |
| Less weight loss than expected | 4.2 ± 0.9 | 4.1 ± 0.9 | 0.971 | 3.1 ± 1.1 | 3.0 ± 1.0 | 0.856 | 2.7 ± 1.1 | 2.9 ± 1.0 | 0.569 |
| Recurrent weight gain | 4.3 ± 0.9 | 4.2 ± 1.0 | 0.940 | 3.1 ± 1.2 | 3.1 ± 1.2 | 0.906 | 2.8 ± 1.3 | 3.0 ± 1.1 | 0.720 |
| Reoperation | 4.3 ± 0.8 | 4.0 ± 1.0 | 0.413 | 2.7 ± 1.1 | 2.6 ± 0.9 | 0.985 | 2.8 ± 1.1 | 2.9 ± 0.9 | 0.714 |
| Symptomatic cholelithiasis | 4.1 ± 1.0 | 3.9 ± 1.1 | 0.790 | 2.5 ± 1.1 | 2.7 ± 1.0 | 0.581 | 2.5 ± 1.1 | 2.8 ± 0.9 | 0.464 |
| Complaints of hypoglycemia | 4.1 ± 1.0 | 4.0 ± 1.1 | 0.948 | 2.4 ± 0.8 | 2.7 ± 0.9 | 0.343 | 2.6 ± 1.1 | 2.9 ± 1.0 | 0.434 |
| Diarrhea | 3.9 ± 1.3 | 4.0 ± 1.0 | 0.979 | 2.4 ± 1.1 | 2.8 ± 1.0 | 0.141 | 2.6 ± 1.2 | 2.9 ± 0.9 | 0.346 |
| Deficiency in vitamins and/or minerals | 4.3 ± 0.9 | 4.2 ± 0.9 | 0.760 | 2.8 ± 1.2 | 2.9 ± 1.0 | 0.847 | 2.8 ± 1.2 | 3.0 ± 1.0 | 0.603 |
| *Numbers of responses varying between 37 – 41 (< 30 years) and 99 - 104 (> 50 years) on each domain.  ^×^Adjusted P value (Benjamini-Hochberg correction).  Data expressed as mean ± standard deviation. Validation of the 5-point Likert scale: 1 = not important at all, 2 = not important, 3 = neutral, 4 = important, 5 = very important. GERD = gastroesophageal reflux disease. | | | | | | | | | |

| **Table 9 –** Impact of complications, subgroup analysis based on the presence of comorbidities | | | | | | | | | |
| --- | --- | --- | --- | --- | --- | --- | --- | --- | --- |
|  | **Information** | | | **Anxiety** | | | **Consideration** | | |
|  | **With obesity-related diseases**  N=153* | **Without obesity-related diseases**  N=59* | P value^×^ | **With obesity-related diseases**  N=153* | **Without obesity-related diseases**  N=59* | P value^×^ | **With obesity-related diseases**  N=153* | **Without obesity-related diseases**  N=59* | P value^×^ |
| **Short-term complications (≤ 30 days after surgery)** | | | | | | | | | |
| Bleeding | 4.3 ± 0.9 | 4.4 ± 0.8 | 0.901 | 2.6 ± 1.0 | 2.9 ± 1.0 | 0.351 | 2.7 ± 1.1 | 2.8 ± 1.1 | 0.906 |
| Anastomotic leakage | 4.3 ± 0.9 | 4.4 ± 0.8 | 0.632 | 2.7 ± 1.0 | 2.9 ± 1.0 | 0.424 | 2.7 ± 1.1 | 2.8 ± 1.2 | 0.891 |
| Wound infection | 4.3 ± 0.8 | 4.3 ± 0.9 | 1.0 | 2.6 ± 1.0 | 2.7 ± 1.0 | 0.996 | 2.6 ± 1.1 | 2.6 ± 1.1 | 0.874 |
| Reoperation | 4.1 ± 1.0 | 4.2 ± 0.9 | 0.689 | 2.5 ± 1.0 | 2.7 ± 1.1 | 0.583 | 2.7 ± 1.1 | 2.8 ± 1.2 | 0.939 |
| Pneumonia | 4.0 ± 1.1 | 4.0 ± 1.0 | 1.0 | 2.5 ± 1.0 | 2.4 ± 1.0 | 0.770 | 2.6 ± 1.1 | 2.5 ± 1.2 | 0.839 |
| Thrombosis/ pulmonary embolism | 4.2 ± 1.0 | 4.2 ± 1.0 | 0.995 | 2.7 ± 1.0 | 2.7 ± 1.0 | 0.989 | 2.7 ± 1.1 | 2.7 ± 1.2 | 0.996 |
| Readmission | 4.1 ± 1.1 | 4.1 ± 1.0 | 0.962 | 2.6 ± 1.0 | 2.7 ± 1.2 | 0.740 | 2.7 ± 1.1 | 2.6 ± 1.3 | 0.942 |
| Constipation | 4.0 ± 1.1 | 4.1 ± 0.9 | 0.896 | 2.5 ± 1.0 | 2.5 ± 1.1 | 0.906 | 2.6 ± 1.1 | 2.5 ± 1.2 | 0.761 |
| Difficulties with eating and drinking/ nausea and vomiting | 4.2 ± 1.0 | 4.3 ± 0.9 | 0.772 | 2.8 ± 1.1 | 3.2 ± 1.1 | 0.063 | 2.8 ± 1.1 | 2.9 ± 1.2 | 0.822 |
| **Long-term complications (> 2 years after surgery)** | | | | | | | | | |
| Internal herniation | 4.1 ± 1.0 | 4.4 ± 0.9 | 0.137 | 2.9 ± 1.0 | 3.2 ± 0.9 | 0.099 | 2.9 ± 1.1 | 3.1 ± 1.1 | 0.433 |
| Gastric ulcer | 4.1 ± 1.0 | 4.2 ± 1.0 | 0.709 | 2.7 ± 0.9 | 2.9 ± 0.9 | 0.409 | 2.7 ± 1.0 | 2.8 ± 1.1 | 0.977 |
| GERD | 4.1 ± 1.0 | 4.1 ± 1.0 | 0.983 | 2.9 ± 1.0 | 2.9 ± 1.0 | 0.992 | 2.8 ± 1.1 | 2.8 ± 1.1 | 0.890 |
| Dumping | 4.2 ± 0.9 | 4.3 ± 0.9 | 0.812 | 2.9 ± 1.1 | 3.2 ± 1.0 | 0.379 | 2.9 ± 1.0 | 3.0 ± 1.2 | 0.761 |
| Malnutrition | 4.1 ± 1.0 | 4.1 ± 1.0 | 0.896 | 2.6 ± 1.0 | 2.5 ± 1.1 | 0.765 | 2.7 ± 1.0 | 2.7 ± 1.2 | 0.885 |
| Underweight | 4.0 ± 1.0 | 4.0 ± 1.1 | 1.0 | 2.6 ± 1.0 | 2.4 ± 1.0 | 0.576 | 2.7 ± 1.0 | 2.6 ± 1.1 | 0.920 |
| Less weight loss than expected | 4.1 ± 0.9 | 4.1 ± 1.0 | 0.982 | 3.0 ± 1.0 | 3.2 ± 1.1 | 0.422 | 2.8 ± 1.1 | 2.8 ± 1.2 | 1.0 |
| Recurrent weight gain | 4.2 ± 0.9 | 4.2 ± 0.9 | 0.824 | 3.0 ± 1.1 | 3.2 ± 1.2 | 0.631 | 2.9 ± 1.2 | 2.9 ± 1.2 | 0.932 |
| Reoperation | 4.0 ± 1.1 | 4.1 ± 1.1 | 0.784 | 2.6 ± 1.0 | 2.8 ± 1.0 | 0.391 | 2.9 ± 1.1 | 2.8 ± 1.0 | 0.938 |
| Symptomatic cholelithiasis | 4.0 ± 1.1 | 3.9 ± 1.1 | 0.982 | 2.6 ± 1.1 | 2.7 ± 1.1 | 0.805 | 2.7 ± 1.1 | 2.7 ± 1.1 | 0.835 |
| Complaints of hypoglycemia | 4.0 ± 1.1 | 4.0 ± 1.2 | 0.996 | 2.7 ± 1.0 | 2.6 ± 0.9 | 0.892 | 2.8 ± 1.0 | 2.7 ± 1.0 | 0.991 |
| Diarrhea | 4.0 ± 1.0 | 3.8 ± 1.2 | 0.661 | 2.8 ± 1.0 | 2.7 ± 1.0 | 0.814 | 2.8 ± 1.0 | 2.8 ± 1.1 | 0.988 |
| Deficiency in vitamins and/or minerals | 4.2 ± 0.9 | 4.2 ± 1.0 | 0.872 | 2.8 ± 1.0 | 2.9 ± 1.1 | 0.976 | 2.9 ± 1.1 | 2.9 ± 1.2 | 1.0 |
| *Numbers of responses varying between 142 - 152 (with comorbidities) and 54 - 59 (without comorbidities) on each domain. ^×^Adjusted P value (Benjamini-Hochberg correction).  Data expressed as mean ± standard deviation. Validation of the 5-point Likert scale: 1 = not important at all, 2 = not important, 3 = neutral, 4 = important, 5 = very important. GERD = gastroesophageal reflux disease. | | | | | | | | | |
